# Sustainable Room-Temperature Sol–Gel Synthesis of Mesoporous Silica Nanoparticles from Sodium Silicate Using Ascorbic Acid and Nonionic Surfactants for Amoxicillin Removal from Water

**DOI:** 10.3390/nano16130799

**Published:** 2026-06-27

**Authors:** Manal A. Almalki, Obaid A. Alharbi, Sultan K. Alharbi, Bandar R. Alsehli, Khaled A. Thumayri, Khaled M. AlMohaimadi, Yassin T. H. Mehdar, Awadh O. AlSuhaimi, Belal H. M. Hussein

**Affiliations:** 1 Chemistry Department, Faculty of Science, Taibah University, Madinah Al-Munawarah 41321, Saudi Arabia; mmalky@taibahu.edu.sa (M.A.A.); sbdrani@taibahu.edu.sa (S.K.A.); bshle@taibahu.edu.sa (B.R.A.); kthumairi@taibahu.edu.sa (K.A.T.); ymehdar@taibahu.edu.sa (Y.T.H.M.); 2 Water Management & Treatment Technologies Institute, Sustainability and Environment Sector, King Abdulaziz City for Science and Technology (KACST), Riyadh 12354, Saudi Arabia; oaharbi@kacst.gov.sa; 3 Ministry of Education, Directorate General of Education, Madinah Al-Munawarah 42314, Saudi Arabia; khaled-mohaimadi@hotmail.com; 4 Chemistry Department, Faculty of Science, Suez Canal University, Ismailia 41522, Egypt; belalhussein102@yahoo.com

**Keywords:** mesoporous silica nanoparticles, green synthesis, sodium silicate, room-temperature sol–gel, ascorbic acid, nonionic surfactants, amoxicillin adsorption, pharmaceutical pollutants, water remediation, sustainable nanotechnology

## Abstract

Mesoporous silica nanoparticles (MSNs) are promising nanomaterials for many applications, including water remediation, owing to their high surface area, tunable mesoporosity, and modifiable silanol-rich surfaces. However, their conventional synthesis often relies on costly tetraethyl orthosilicate (TEOS), cationic surfactants, organic solvents, and energy-intensive hydrothermal processing. Herein, a facile sustainable room-temperature sol–gel route is reported using inexpensive sodium silicate as the silica source, L-ascorbic acid as a mild biodegradable acid catalyst, and a binary nonionic surfactant system, Triton X-100/polysorbate 80, as the structure-directing template. The method replaces alkoxysilanes and hazardous cationic templates and eliminates external heating. It enables the production of uniform spherical MSNs with a locally ordered mesoporous structure, high specific surface area up to 551.5 m^2^ g^−1^, and large pore volume up to 1.98 cm^3^ g^−1^. The adsorption capability of the optimized MSNs as nano-adsorbents was demonstrated using amoxicillin (AMX) as a model pharmaceutical contaminant. The optimized sample showed maximum AMX uptake at pH 5.0, followed pseudo-second-order kinetics, and fitted the Langmuir isotherm with a monolayer capacity of 91.3 mg g^−1^. In spiked water matrices, the optimized MSNs recovered 88.5% and 84.4% of AMX from tap water spiked at 10 and 50 mg L^−1^, respectively, and 83.5% and 81.0% from synthetic municipal wastewater spiked at the same concentrations, with RSD values below 5%. The adsorbent further retained 94% of its initial capacity after five adsorption–desorption cycles. This work establishes a scalable green route for producing high-quality MSNs and demonstrates the feasibility of the resulting silanol-rich mesoporous nano-adsorbents for pharmaceutical micropollutant removal, while also indicating their potential suitability as carrier platforms for drug-delivery applications.

## 1. Introduction

Silica-based nanomaterials have progressed through increasingly precise control over structure, surface chemistry, and functional behavior across multiple length scales [1,2]. Early studies on colloidal silica showed that hydrolysis and condensation reactions control how particles nucleate, grow, and remain stable in water. That understanding enables the production of uniform spherical particles with adjustable sizes and reliable colloidal stability [3,4]. The finding of these studies formed the basis of sol–gel chemistry. Nonetheless, the materials produced in this early stage were largely dense and nonporous, which limited accessible surface area and slowed mass transport within the particles.

The Stöber method provides a robust route to monodisperse spherical silica particles through controlled hydrolysis and condensation of alkoxysilane precursors in alcoholic media [5]. Fine-tuning of reaction parameters enables reproducible control over particle size, morphology, and dispersity. However, the resulting particles are typically dense solids with limited accessible internal porosity. Consequently, specific surface area remains relatively low and intraparticle diffusion is restricted, limiting performance in applications that depend on high interfacial exposure or rapid mass transport.

This limitation was addressed in the early 1990s with the development of ordered mesoporous silicas, including MCM-41 and the SBA family. Surfactant-templated self-assembly enabled the formation of periodic pore networks with adjustable pore diameters and exceptionally high surface areas [6,7]. This structural design significantly expanded the use of silica materials in catalysis, separation processes, and controlled release systems [8]. Nevertheless, these materials were predominantly obtained as bulk powders or irregular aggregates, which compromised colloidal stability and restricted their effectiveness in nanoscale interfacial environments.

A further advance emerged from integrating the structural uniformity of Stöber-derived spheres with the ordered mesoporosity of surfactant-templated silica. This led to mesoporous silica nanoparticles (MSNs), which combine colloidal monodispersity with an internally ordered pore architecture within a single nanoscale entity [9,10,11]. MSNs therefore exhibit both high surface area and large pore volume alongside excellent dispersibility in solution, making them particularly suitable for adsorption-driven environmental applications. In addition, the abundance of surface silanol groups facilitates straightforward post-synthetic functionalization, enabling tailored binding properties and controlled release behavior [11,12,13].

Despite these advantages, many conventional MSN syntheses remain energy- and reagent-intensive. Standard routes often use alkoxysilane precursors and cationic surfactants such as cetyltrimethylammonium bromide or chloride (CTAB/CTAC). They may also require hydrothermal aging at 80–130 °C to consolidate the framework and improve mesoscale ordering [14,15,16]. Such thermal steps increase energy consumption and processing time, reducing sustainability and scalability [17]. In addition, CTAB/CTAC and related cationic surfactants are toxic, poorly biodegradable, and commonly require harsh removal protocols that can damage surface silanols and reduce functional performance [18]. These limitations highlight the need for MSN synthesis strategies that minimize energy-intensive thermal treatments, avoid the use of persistent cationic surfactants, and maintain precise control over particle morphology and mesoporous structure. The growing interest in this direction is reflected in recent studies focused on improving the sustainability and scalability of mesoporous silica production [19], as well as in efforts to advance green silica synthesis within a circular-economy framework [20,21].

Within a green-chemistry framework, an ideal MSN synthesis should use low-cost and abundant precursors, employ water as the principal solvent, operate at ambient temperature, and rely on biodegradable catalysts and templates that can be removed under mild conditions. Sodium silicate (water glass), an industrially abundant alkali silicate, is an attractive alternative to TEOS from economic and environmental perspectives. L-Ascorbic acid, a food-grade biodegradable weak acid, can serve as a mild proton donor that moderates silicate hydrolysis-condensation kinetics without generating persistent toxic by-products. Nonionic surfactants such as Triton X-100 and polysorbate 80 (Tween 80) are widely used in food and pharmaceutical formulations and assemble into micellar templates through hydrogen bonding rather than electrostatic interactions, facilitating mild template removal.

This work presents an integrated room-temperature sol–gel strategy that combines sodium silicate as a water-soluble silica source, L-ascorbic acid as a biodegradable catalyst, and a binary polysorbate 80/Triton X-100 nonionic surfactant system as the structure-directing template. The key advance is the simultaneous elimination of alkoxysilanes, cationic surfactants, and elevated-temperature processing while maintaining control over nanoparticle size, mesopore ordering, and textural properties. Unlike electrostatically driven CTAB/CTAC-templated systems, the nonionic mixed-micelle approach enables mesostructure formation under mild conditions and facilitates surfactant removal. The resulting MSNs were evaluated as nanosorbents for amoxicillin (AMX) removal from aqueous systems, linking the synthetic advance to environmentally relevant water-treatment performance through kinetic and equilibrium adsorption descriptors.

## 2. Materials and Methods

### 2.1. Materials

Sodium silicate solution (27–35 wt% SiO_2_, commercial grade) was kindly provided by Arjan Industrial Company (Riyadh, Saudi Arabia). Polysorbate 80 (≥99%), Triton X-100 (laboratory grade), L-ascorbic acid (≥99.5%), and amoxicillin trihydrate (AMX, ≥98%) were purchased from Sigma-Aldrich (St. Louis, MO, USA). Absolute ethanol (≥99.8%) was obtained from Sadara Chemical Company (Jubail Industrial City, Saudi Arabia). Ultrapure water (18.2 MΩ·cm) prepared with a Milli-Q purification system (Millipore, Darmstadt, Germany) was used throughout. All reagents were used as received without further purification.

### 2.2. Synthesis of Mesoporous Silica Nanoparticles

MSNs were synthesized using a one-pot room-temperature sol–gel procedure developed in this work, with sodium silicate as the silica precursor, L-ascorbic acid as a mild acid catalyst, and a Triton X-100/polysorbate 80 binary surfactant system as the structure-directing agent. In a typical synthesis, ethanol (25 mL) and deionized water (50 mL) were mixed under continuous stirring at room temperature, and L-ascorbic acid (2.00 g) was dissolved to obtain a homogeneous reaction medium. Triton X-100 (0.20 mL; 0.33 mmol; 2.65 mM), at a concentration above its critical micelle concentration (CMC approximately 0.22 mM) [22], was then added, followed by polysorbate 80 (CMC approximately 0.012 mM) [23]. A freshly prepared 5 wt% aqueous sodium silicate solution (50 mL) was subsequently introduced dropwise under constant stirring to promote controlled hydrolysis and condensation of silicate species. Polysorbate 80 was used at three concentrations (0.005, 0.0135, and 1.35 mM), while all other parameters were kept constant; the resulting samples were denoted MSN-1, MSN-2, and MSN-3, respectively. The reaction mixture was stirred for 90 min and then aged statically at room temperature overnight. The resulting solids were collected by centrifugation (4000 rpm, 10 min), washed sequentially with ethanol and deionized water, dried at 80 °C for 8 h, and calcined at 550 °C in air to remove the surfactant template. The calcined MSNs were stored in a desiccator until use.

To remove residual sodium from the sodium silicate precursor, the calcined material was subjected to a mild acid-driven cation-exchange step. Briefly, 1.0 g of calcined MSN-3 powder was suspended in 100 mL of 0.1 M HCl and stirred at room temperature (300 rpm) for 4 h. During this step, Na^+^ ions associated with deprotonated silanol groups (Si-O^−^Na^+^) were displaced by H^+^ to regenerate free silanols (Si-OH) [24]. The protonated solid was recovered by centrifugation (4000 rpm, 10 min) and washed repeatedly with deionized water until the supernatant reached constant neutral pH and gave a negative AgNO_3_ spot test for chloride. The exchanged powder was then dried at 80 °C for 12 h and stored in a desiccator. The effectiveness of this purification step is shown by EDS and elemental mapping which indicate near-complete removal of the residual sodium signal.

### 2.3. Characterization

Powder X-ray diffraction (XRD) patterns were collected on a Siemens D5005 diffractometer (Siemens AG, Munich, Germany) operated with Cu Kα radiation (λ = 1.5406 Å) at 40 kV and 30 mA, in both small-angle and wide-angle modes. Nitrogen adsorption–desorption isotherms were recorded at 77 K on a Micromeritics ASAP 2010 M analyzer (Micromeritics, Norcross, GA, USA). Specific surface areas were calculated by the Brunauer–Emmett–Teller (BET) method in the relative pressure range P/P_0_ = 0.05–0.45, pore size distributions were obtained from the Barrett–Joyner–Halenda (BJH) model, and total pore volumes were estimated at P/P_0_ = 0.99. Fourier-transform infrared (FTIR) spectra were acquired using a JASCO FT/IR-420 spectrometer (JASCO Corporation, Tokyo, Japan) by the KBr pellet method. Surface morphology was examined by scanning electron microscopy (SEM) on a JEOL JSM-6300 instrument (JEOL Ltd., Tokyo, Japan) at 20–30 kV; samples were sputter-coated with platinum before imaging. Energy-dispersive X-ray spectroscopy (EDS) and elemental mapping were performed in conjunction with SEM. Particle size distributions were derived from SEM micrographs using ImageJ/Fiji software (v1.54f; National Institutes of Health, Bethesda, MD, USA (https://imagej.net/)). Solution pH values were monitored with a HI2020-02 pH meter (HANNA Instruments, Smithfield, VA, USA), and ultraviolet–visible (UV-Vis) absorption spectra were recorded on a Shimadzu UV-1800 spectrophotometer (Shimadzu Corporation, Kyoto, Japan).

### 2.4. Adsorption Studies

The synthesized MSNs were evaluated as nano-adsorbents for AMX removal from water to demonstrate the practical value of the developed green synthesis route. AMX was selected as a representative pharmaceutical micropollutant because antibiotics are commonly detected in aquatic environments and may persist after conventional wastewater treatment. This application is justified by the high surface area, interconnected mesoporous structure, and silanol-rich surface of MSNs, which provide accessible adsorption sites, facilitate AMX diffusion into the pore network, and promote hydrogen-bonding and pH-dependent electrostatic interactions with polar AMX functional groups. Therefore, adsorption experiments were conducted to assess pH effect, kinetics, equilibrium capacity, reusability, and performance in spiked water matrices. All equilibrium and kinetic measurements were performed at a single ambient temperature (298 K), which has been selected to reflect typical surface-water and treatment conditions and to remain consistent with the room-temperature character of the synthesis.

#### 2.4.1. Equilibrium Isotherm Studies

Equilibrium adsorption experiments were performed by contacting 100 mg of MSNs with 100 mL of AMX solution at initial concentrations of 20–250 mg L^−1^. The suspensions were agitated at 200 rpm in a thermostatically controlled shaker at room temperature with the pH adjusted to 5.0. After equilibration, the mixtures were centrifuged, and residual AMX in the supernatant was quantified by UV-Vis spectroscopy at 228 nm using a pre-established calibration curve [24].

The equilibrium adsorption capacity (*q_e_*, mg g^−1^) and adsorption efficiency (*η*, %) were calculated using Equations (1) and (2):

*q_e_* = (*C*_0_ − *C_e_*) × *V*/*m* (1)

*η* (%) = [(*C*_0_ − *C_e_*)/*C*_0_] × 100 (2)

where *C*_0_ and *C_e_* (mg L^−1^) are the initial and equilibrium AMX concentrations, *V* (L) is the solution volume, and *m* (g) is the adsorbent mass. Equilibrium data were analyzed using the Langmuir and the Freundlich isotherm models to differentiate monolayer adsorption on energetically uniform sites from heterogeneous adsorption on the mesoporous surface.

#### 2.4.2. Adsorption Kinetic Studies

Kinetic experiments were performed by dispersing 100 mg of MSN-3 in 100 mL of 100 mg L^−1^ AMX solution at pH 5.0 and 25 °C under continuous agitation. At predefined time intervals (0–240 min), 1 mL aliquots were withdrawn, immediately centrifuged, and analyzed by UV-Vis spectroscopy. The adsorption capacity at time *t*, *q_t_* (mg g^−1^), was calculated using Equation (3):

*q_t_* = (*C*_0_ − *C_t_*) × *V*/*m* (3)

where *C_t_* (mg L^−1^) is the AMX concentration at time *t*. The kinetic data were analyzed using the pseudo-first-order (PFO) and pseudo-second-order (PSO) models, as detailed in Section 3.2.2.

### 2.5. Point of Zero Charge (pH_PZC_) Determination

The point of zero charge (pH_PZC_) of MSN-3, defined as the pH at which the net surface charge is zero, was determined by the salt-addition method [9]. Aliquots of 0.01 M NaCl (10 mL) were adjusted to initial pH values (pH_i_) between 3.0 and 9.0 using 0.1 M HCl or NaOH. MSNs (50 mg) were added to each flask and shaken on an IKA KS 3000i platform (IKA-Werke, Königswinter, Germany) at room temperature for 12 h. The suspensions were then centrifuged, and the final pH (pHf) of the supernatant was measured using the HI2020-02 pH meter. The pH_PZC_ was identified as the intersection of the pH_i_-pHf curve with the pH_i_ = pHf line.

## 3. Results and Discussion

### 3.1. Synthesis Strategy and Characterization of MSNs

The proposed sol–gel strategy in this work produced mesoporous silica nanoparticles with well-defined morphology, high specific surface area, and accessible pore networks under ambient conditions using sodium silicate and biodegradable nonionic surfactants. This route shows that mesostructured silica frameworks can be obtained without alkoxysilane precursors, elevated temperatures, or hydrothermal aging, which are often used in conventional sol–gel synthesis [25].

The formation of mesostructure is primarily controlled by the interplay between silicate condensation kinetics and surfactant self-assembly. Sodium silicate supplies reactive silicate oligomers that undergo gradual hydrolysis and condensation under the mild acidic conditions provided by L-ascorbic acid. The weak acidity is thought to moderate nucleation, thereby suppressing uncontrolled precipitation and favoring uniform silica deposition at the organic–inorganic interface [26,27]. In parallel, polysorbate 80 and Triton X-100 co-assemble into mixed micelles that function as soft templates guiding silica growth. The silicate species condense around these micellar structures, forming an ordered inorganic framework. Stabilization of the silica–surfactant interface is facilitated by hydrogen bonding between surface silanol (Si–OH) groups and the ethylene oxide chains of the nonionic surfactants. Following template removal, the hydrophobic micellar domains define the resulting mesoporous architecture.

**Figure 1:**
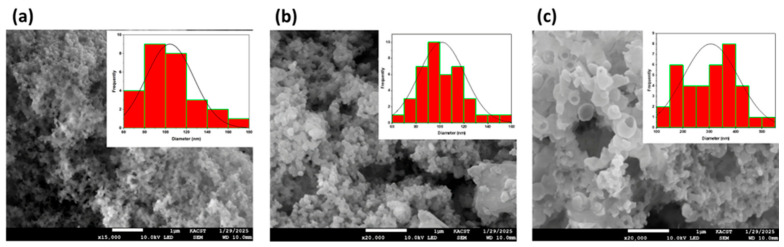
SEM micrographs of MSNs prepared with different polysorbate 80 concentrations, with particle-size distributions shown as insets: (**a**) MSN-1, 0.005 mM; (**b**) MSN-2, 0.0135 mM; and (**c**) MSN-3, 1.35 mM.

Systematic variation in polysorbate 80 concentration, the primary structure-directing surfactant, across values below and above its CMC (approximately 0.012 mM) enabled control over particle size. As shown in Figure 1, increasing surfactant concentration from 0.005 to 1.35 mM produced a progressive increase in MSN diameter from approximately 100 to 335 nm. MSN-1, MSN-2, and MSN-3 exhibited mean diameter of approximately 100, 105, and 335 nm, respectively. The corresponding size distributions are presented as the histograms inset in Figure 1; their full widths are broader than these mean values alone convey and reflect the inherent polydispersity of the samples. SEM further revealed fractured spheres with clearly defined internal cavities, indicating hierarchical core–shell architectures composed of mesoporous silica shells around hollow cores. Hollow-core diameters ranged from 135 to 250 nm, and shell thickness remained relatively uniform at 30–55 nm. The contrast between the core and shell arises from the lower electron density of the void interior relative to the silica framework and is consistent with surfactant-templated systems, in which polysorbate 80 promotes micelle formation followed by preferential silica condensation around removable templates [28,29].

**Figure 2:**
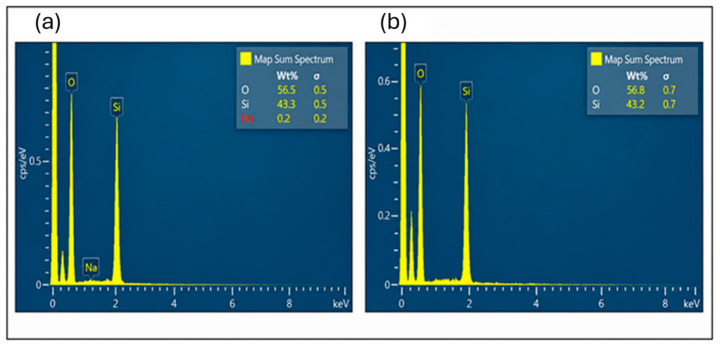
EDS spectra of MSN-3 (**a**) before and (**b**) after ion-exchange treatment of the sodium-silicate-derived material.

Energy-dispersive X-ray spectroscopy (EDS) was used to monitor the elemental composition of MSN-3 before and after cation-exchange purification. As shown in Figure 2, the sodium signal observed in the as-prepared sample was effectively eliminated after ion exchange, indicating efficient removal of residual sodium species from the silica framework. The post-exchange composition was dominated by silicon and oxygen, with weight percentages of 43.2% (Si) and 56.8% (O), corresponding to atomic percentages of 31.2% and 68.8%, respectively, which agrees with the expected composition of SiO_2_.

**Figure 3:**
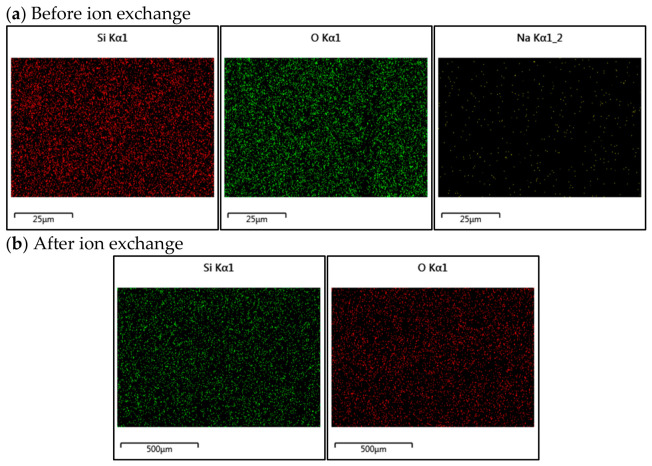
EDS elemental mapping of MSN-3 (**a**) before and (**b**) after ion-exchange treatment of the sodium-silicate-derived material.

Elemental mapping (Figure 3) further confirms the compositional homogeneity of the MSNs. The Si and O distributions closely follow the particle morphology and indicate uniform incorporation of both elements throughout the silica framework. Only a trace amount of sodium (0.2 wt%) is detected in the as-prepared sample (Figure 3a), attributable to residual precursor; this signal is essentially absent after ion exchange (Figure 3b), supporting the formation of chemically clean MSNs.

**Figure 4:**
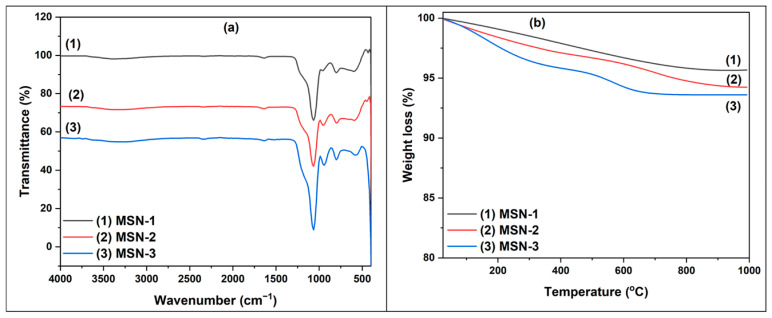
(**a**) FTIR spectrum and (**b**) TGA profile of MSNs after calcination at 550 °C to remove the Triton X-100/polysorbate 80 template.

FTIR spectroscopy was used to elucidate the chemical structure of the calcined MSNs (Figure 4a). The characteristic absorptions appeared at 1082 cm^−1^ (asymmetric Si-O-Si stretching), 801 cm^−1^ (symmetric Si-O-Si stretching), and 465 cm^−1^ (Si-O-Si bending), confirming formation of a condensed silica network [30,31]. Additional bands at 3391 cm^−1^ (O-H stretching) and 1638 cm^−1^ (O-H bending) were assigned to surface silanols and physisorbed water [32], while the band at 946 cm^−1^ confirmed Si-OH vibrations characteristic of mesoporous silica. The absence of strong C-H bands near 2800–3000 cm^−1^ indicates effective removal of the surfactant template after calcination at 550 °C. Together, these spectral features confirm successful synthesis of silanol-rich MSNs, an important attribute for adsorption and post-functionalization applications.

Thermogravimetric analysis (TGA) of the calcined MSNs (Figure 4b) demonstrated excellent thermal stability. The initial mass loss of 2–3% between 25 and 150 °C is attributed to desorption of physisorbed and capillary-condensed water within the mesopores [33,34], consistent with the hydrophilic, silanol-rich character of mesoporous silica reported by Wu and Navrotsky [35]. From 180 to 900 °C, only a minor additional weight loss (3–6%) was observed, corresponding to gradual dehydroxylation (Si-OH + HO-Si -> Si-O-Si + H_2_O) and trace residual organics. The residual mass above 600 °C (>90%) confirms high inorganic content and structural robustness after calcination.

**Figure 5:**
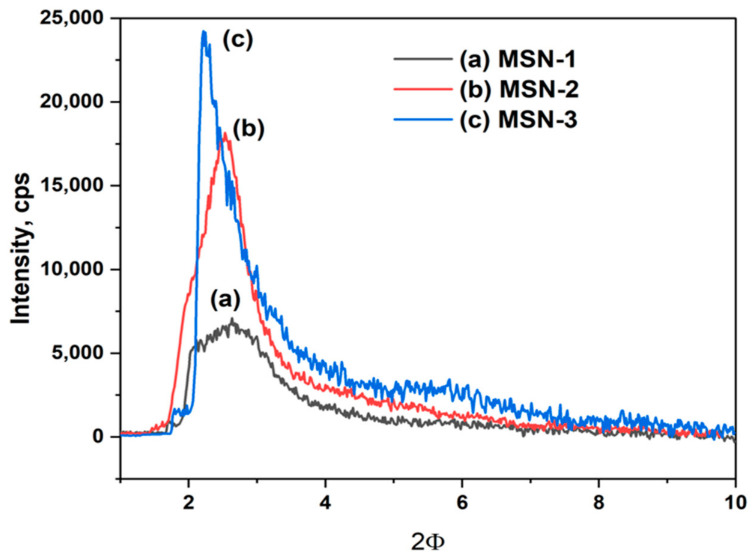
Small-angle XRD patterns of MSNs prepared at different polysorbate 80 concentrations.

**Figure 6:**
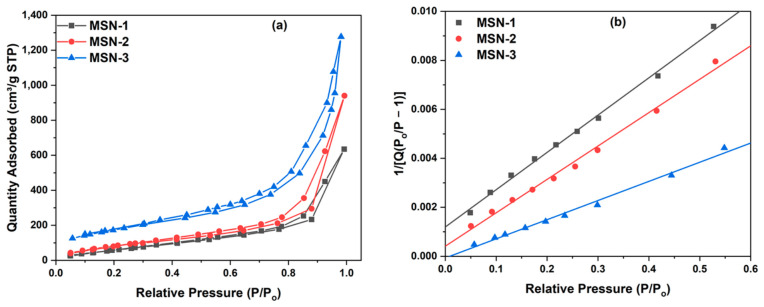
(**a**) N_2_ adsorption–desorption isotherms of MSNs and (**b**) corresponding linearized plots in the relative-pressure range used for BET analysis.

Small-angle X-ray diffraction (SAXRD) was used to examine mesoscale ordering in MSNs. As shown in Figure 5, all samples displayed a prominent reflection in the 2θ range of 1.8–2.5°, which corresponds to a characteristic, repeating pore-to-pore correlation distance. A single low-angle reflection of this kind indicates short-range (local) ordering of the mesopores but is not sufficient on its own to establish a long-range two-dimensional hexagonal (p6mm) mesostructure, the unambiguous assignment of which would require higher-order reflections such as (110) and (200). In the Triton X-100/polysorbate 80 binary system, the intensity of this peak increased with polysorbate 80 concentration from 0.005 to 1.35 mM, reaching maximum sharpness and intensity at 1.35 mM. This trend indicates improved pore ordering driven by optimized micellar templating. At polysorbate 80 concentrations below 0.01 mM, the low-angle peak broadened and shifted to higher 2θ angles, indicating lower periodicity and smaller pore dimensions. As noted above, these higher-order reflections were not detected. This absence, together with the H3-type hysteresis loops and the lack of a sharp pore-condensation step in the N_2_ isotherms (Figure 6), and the fact that Triton X-100 assembles mainly into spherical or ellipsoidal rather than long cylindrical micelles, indicates that the present materials possess a locally ordered but globally disordered (wormhole-like) mesopore network rather than an ordered hexagonal array of cylindrical channels [36]. At low polysorbate 80 levels, Triton X-100-dominated spherical micelles produce less ordered frameworks, whereas an optimal polysorbate 80/Triton X-100 molar ratio of 0.50 yields the most uniform mesopore spacing, as indicated by the narrowest and most intense low-angle reflection. This behavior underscores the role of mixed-micelle morphology in directing silica condensation [25,37].

Nitrogen adsorption–desorption isotherms measured at 77 K (Figure 6), along with the derived textural parameters (Table 1), confirm that all samples exhibit a well-developed mesoporous structure. Each material displays a Type IV isotherm with an H3 hysteresis loop (IUPAC classification), which is characteristic of slit-like mesopores and interparticle voids arising from particle aggregation [38]. With increasing polysorbate 80 concentrations (from 0.005 mM (MSN-1) to an intermediate value (MSN-2) and further to 1.35 mM (MSN-3)) the porosity improves systematically. The BET surface area increases from 264.9 m^2^ g^−1^ (MSN-1) to 309.8 m^2^ g^−1^ (MSN-2) and 551.5 m^2^ g^−1^ (MSN-3), while the total pore volume rises correspondingly from 0.98 to 1.45 and 1.98 cm^3^ g^−1^, respectively. In contrast, the BJH pore diameter exhibits a non-linear trend (14.83 → 18.78 → 14.33 nm), suggesting a shift in the templating mechanism. At the lowest polysorbate 80 content, Triton X-100-rich micelles dominate, producing relatively small mesopores. At intermediate concentration, partial incorporation of polysorbate 80 into the mixed micelles increases their hydrodynamic size, leading to pore expansion. At the highest concentration (MSN-3), the system favors a higher number density of more uniform mixed micelles, which generates a denser mesoporous network with thinner pore walls and a larger specific surface area, albeit with a reduction in the average pore diameter. Accordingly, MSN-3 is identified as the optimal nanoparticle: it possesses the highest surface area and pore volume, while its mesopore diameter (14.33 nm) remains substantially larger than the molecular size of amoxicillin (~1.5 nm), ensuring efficient diffusion and accessibility.

### 3.2. Adsorption Performance for Amoxicillin Removal

The synthesized MSNs were evaluated as nanosorbents for the extraction/removal of AMX from water matrix. Their nanoscale dimensions, high surface area, hierarchical mesoporous architecture, and abundant surface silanol groups can promote adsorption of polar pharmaceutical contaminants through electrostatic interactions and hydrogen bonding [30,38]. AMX was selected as a representative antibiotic pollutant because it is frequently detected in aquatic environments and is not fully removed by conventional wastewater-treatment processes [39,40,41]. AMX is a β-lactam antibiotic with three pKa values (approximately 2.4, 7.4, and 9.6), corresponding to the carboxylic acid, amine, and phenolic hydroxyl groups, respectively; its pH-dependent speciation therefore strongly affects interactions with charged silica surfaces.

#### 3.2.1. Optimization of Solution pH

**Figure 7:**
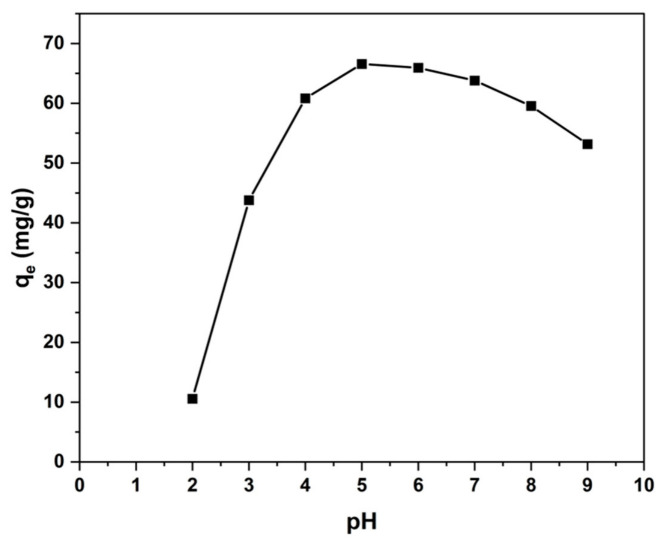
Effect of solution pH on AMX adsorption capacity of MSN-3 (298 K, initial AMX concentration 100 mg L^−1^, equilibrium time 4 h).

**Figure 8:**
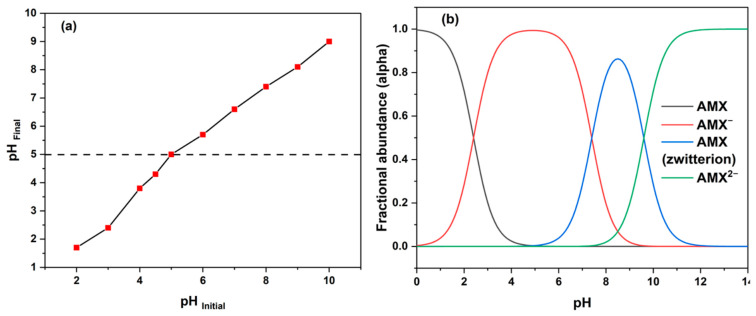
(**a**) Point of zero charge (pH_PZC_) of MSN-3 and (**b**) pH-dependent speciation distribution of AMX.

The pH of the solution governs both the surface charge of silica and the speciation of AMX. Therefore, it was optimized prior to kinetic and equilibrium studies. As shown in Figure 7, the adsorption efficiency of AMX increased with pH, reaching a maximum at pH 5.0. This value closely matches the experimentally determined point of zero charge (pHPZC) of MSN-3 (approximately 5.0; Figure 8a). At this pH, the silica surface exhibits a mixture of protonated (Si-OH_2_^+^) and deprotonated (Si-O^−^) silanol groups, corresponding to the transition region around the pHPZC. Simultaneously, AMX exists predominantly in its zwitterionic form, featuring both a negatively charged carboxylate and a positively charged amino group (Figure 8b) [42]. Under these conditions, the zwitterionic AMX can engage in complementary electrostatic interactions with oppositely charged surface sites (i.e., –COO^−^ with Si-OH_2_^+^, and –NH_3_^+^ with Si-O^−^), in addition to hydrogen bonding within the mesoporous framework. This combination maximizes AMX uptake. Comparable pH-dependent adsorption behavior has been reported for AMX and other β-lactam antibiotics on silica-based adsorbents [39,43,44]. Consequently, pH 5.0 was selected as the optimal working pH for all subsequent adsorption experiments.

#### 3.2.2. Adsorption Kinetics

**Figure 9:**
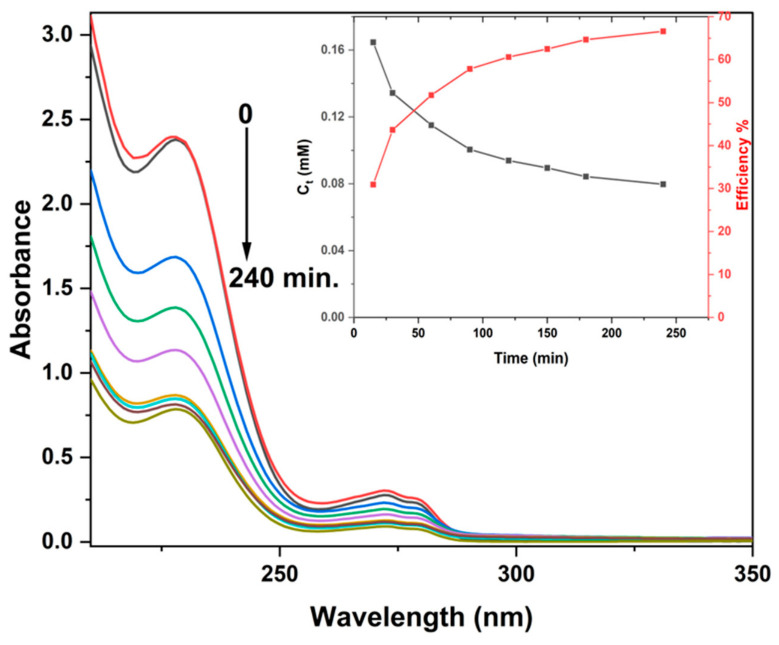
Time-resolved UV-Vis spectra of AMX during adsorption onto MSN-3. Inset: residual AMX concentration and adsorption efficiency as a function of time (298 K, initial AMX concentration 100 mg L^−1^, equilibrium time 4 h).

The effect of contact time on the adsorption of AMX onto the nanosorbent (i.e., MSN-3) was investigated at the optimized pH to elucidate uptake dynamics and identify the controlling mechanism. As shown in Figure 9, the adsorption proceeded rapidly during the initial stage, reaching approximately 66% of the equilibrium uptake within the first 90 min, followed by a slower approach to equilibrium near 240 min. This two-stage profile is characteristic of mesoporous nanosorbents, where fast external-surface adsorption is followed by gradual intraparticle diffusion and saturation of internal active sites [39]. Adsorption kinetics were quantified by fitting the experimental data using the pseudo-first-order (PFO) model of Lagergren and the pseudo-second-order (PSO) model [45,46]. The PSO rate equation is commonly attributed to Ho and McKay; however, its non-linear form was first introduced by Blanchard et al. [47], while Ho and McKay later developed a widely used linearized version. Both models are empirical, and a good statistical fit does not, on its own, confirm a specific adsorption mechanism [48,49]. These models are widely used to distinguish diffusion-controlled uptake from adsorption governed by surface interactions; the kinetic equations and fitting parameters are summarized in Table 2.

Table 2 presents the kinetic parameters describing AMX adsorption onto MSN-3 based on the pseudo-first-order (PFO) and pseudo-second-order (PSO) models. Obviously, the PFO model provides a reasonable but comparatively weaker representation of the experimental data (R^2^ = 0.937), thereby it yields an equilibrium adsorption capacity of 58.2 mg g^−1^ and a rate constant *k*_1_ of 0.018 min^−1^. By contrast, the PSO model exhibits an excellent fit (R^2^ = 0.995) with a markedly lower RMSE (0.89), indicating a substantially improved description of the adsorption kinetics. Importantly, the PSO-predicted equilibrium capacity (66.7 mg g^−1^) aligns closely with the experimentally determined value (≈66.4 mg g^−1^), reinforcing the robustness of this model in capturing the overall uptake behavior. Despite its frequent association with chemisorption processes, the PSO formulation remains empirical and should not be interpreted as definitive mechanistic proof. However, the improved fit relative to the PFO model suggests that adsorption is not governed solely by external film diffusion and instead involves more complex rate-limiting steps. In the context of the optimized particles (MSN-3) surface chemistry and the pH-dependent ionization state of AMX, the uptake process is more consistently explained by synergistic electrostatic interactions and hydrogen bonding with surface silanol groups. Confirmation of a true chemisorption mechanism would require complementary evidence beyond kinetics, such as Elovich modeling or spectroscopic analysis of surface species [40,50].

#### 3.2.3. Adsorption Isotherms

**Figure 10:**
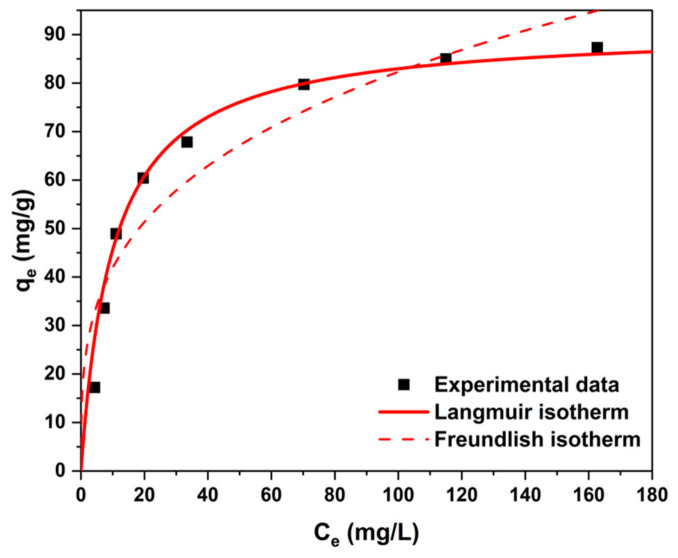
Experimental data and isotherm fits for AMX adsorption on MSN-3 at room temperature and pH 5.0.

Equilibrium isotherms were applied to quantify adsorption capacity and characterize adsorbate-adsorbent interactions at the optimized pH. As shown in Figure 10, the equilibrium adsorption capacity increased with initial AMX concentration and approached a plateau at higher concentrations, indicating saturation of available surface sites. The equilibrium data were analyzed using the Langmuir and Freundlich isotherm models, which describe monolayer adsorption on energetically homogeneous surfaces and adsorption on energetically heterogeneous surfaces, respectively [51,52]. The Freundlich equation does not represent multilayer adsorption; multilayer coverage is instead described by the BET model. The fitting parameters are summarized in Table 3.

The Langmuir isotherm model provided a superior fit to the experimental equilibrium data relative to the Freundlich model, as indicated by higher determination coefficients (R^2^) and lower root-mean-square error (RMSE) values (Table 3). The calculated maximum monolayer adsorption capacity (*q_m_* = 91.3 mg g^−1^) showed excellent agreement with the experimentally observed saturation uptake, consistent with a monolayer adsorption mechanism in which amoxicillin occupies a finite number of energetically homogeneous binding sites, most likely associated with silanol-rich surface functionalities on the aminated MSN [53]. Similar Langmuir-type behavior has been reported for amoxicillin adsorption onto other silica-based nanosorbents [39,50,54], supporting the view that this mechanism is characteristic of aminated silica surfaces under the conditions investigated. The experimental concentration range was deliberately selected to enable reliable construction of equilibrium isotherms and accurate quantification of the maximum adsorption capacity under well-controlled conditions. However, these concentrations are substantially higher than typical environmental levels (µg L^−1^ to ng L^−1^), where amoxicillin uptake must compete with large excesses of dissolved organic matter. Consequently, the performance of the aminated MSN under such ultra-dilute and competitive conditions was not assessed in the present study and represents a key objective for future work, which will require sensitive trace-level analytical methods in complex environmental matrices.

The agreement between the pseudo-second-order kinetic model and the Langmuir isotherm implies that adsorption is primarily governed by surface-controlled interactions at accessible active sites. The uptake process is likely driven by electrostatic interactions between ionized functional groups of AMX and surface silanol groups, in addition to hydrogen bonding contributions that facilitate surface binding. In contrast, intraparticle diffusion and surface heterogeneity may contribute to transport and site distribution effects but are not expected to dominate the equilibrium behavior. The consistency between experimental data and model predictions supports the applicability of mesoporous silica nanoparticles (MSNs) as efficient adsorbents for the removal of antibiotic contaminants from aqueous systems [55,56].

#### 3.2.4. Reusability and Application to Real Water Samples

**Figure 11:**
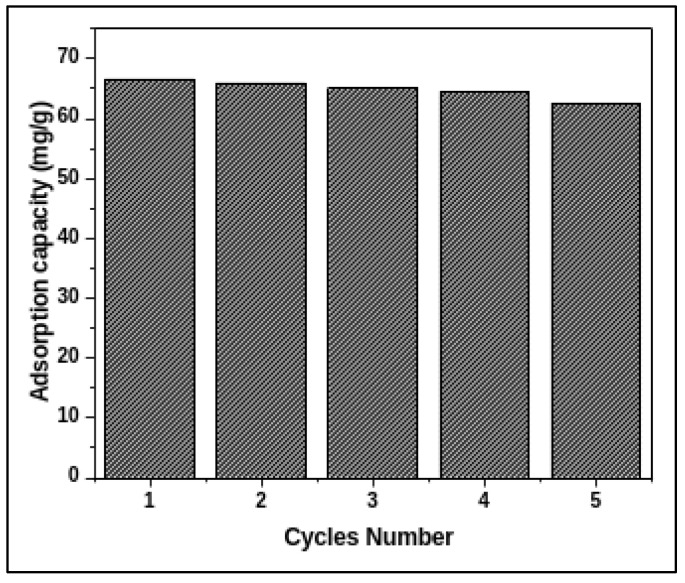
Reusability of MSN-3 over five adsorption–desorption cycles for AMX (initial concentration 100 mg L^−1^, contact time 240 min, adsorbent dose 0.1 g, T = 25.5 °C).

Reusability is a central criterion for sustainable adsorbents because it affects life-cycle cost and environmental footprint. MSN-3 was evaluated over five consecutive adsorption–desorption cycles under the optimized pH conditions to assess operational stability. As shown in Figure 11, MSN-3 retained approximately 94.0% of its initial adsorption capacity after five cycles, indicating good structural integrity and resistance to irreversible fouling. The slight decrease in performance is attributed mainly to partial particle aggregation and unavoidable handling losses during repeated recovery and regeneration rather than degradation of the silica framework. Similar stability has been reported for silica-based nanosorbents used in antibiotic removal [40], supporting the suitability of MSN-3 for repeated use in water-treatment applications.

The adsorptive performance of mesoporous silica nanoparticles (MSNs) as nanosorbents for AMX was evaluated in two distinct aqueous matrices: AMX-spiked tap water and a synthetic municipal wastewater medium prepared according to the protocol described by Shrestha et al. [57]. The latter is a literature-validated municipal-influent surrogate containing organic carbon sources (glucose, soluble starch, and a low level of cellulose), nitrogen sources (urea, ammonium chloride, and peptone), inorganic phosphorus (potassium dihydrogen phosphate), and a balanced background electrolyte (NaCl, MgSO_4_·7H_2_O, CaCl_2_·2H_2_O, FeCl_3_·6H_2_O, KCl, and trace NaHCO_3_ for buffering). The composition delivers a chemical oxygen demand of approximately 500 mg L^−1^, total nitrogen near 50 mg L^−1^, total phosphorus close to 8 mg L^−1^, and circumneutral pH of 7.0–7.5, broadly representative of medium-strength domestic sewage. This matrix exposes MSN-3 to realistic competitive challenges, including dissolved organic matter, ammonium and phosphate species, and divalent cations such as Ca^2+^ and Mg^2+^, which can compete with AMX for silanol-rich adsorption sites. As summarized in Table 4, MSN-3 nevertheless achieved AMX recoveries of 81–89% with relative standard deviations (RSDs) below 5%. Although these recoveries were slightly lower than those obtained in ultrapure water, the material maintained high removal efficiency in the presence of competing inorganic ions and dissolved organic matter typical of real water systems. The preservation of high recoveries across these matrices indicates that the surface-controlled adsorption mechanism identified in model solutions remains operative under environmentally relevant conditions, consistent with previous studies on AMX adsorption by silica-based nanosorbents [39]. Although the synthetic surrogate reproduces the main competitive features of medium-strength sewage, it cannot capture the full complexity of real effluents, including microbial load, heterogeneous natural organic matter, and variable ionic competition. Therefore, the validation with authentic municipal and hospital effluents, where antibiotic residues are most relevant, is therefore identified as an important next step.

## 4. Conclusions

A sustainable, room-temperature sol–gel strategy was successfully established for the synthesis of mesoporous silica nanoparticles (MSNs) using sodium silicate as an inexpensive silica precursor, L-ascorbic acid as a biodegradable catalyst, and nonionic surfactants (Triton X-100 and polysorbate 80) as structure-directing agents. By eliminating alkoxysilane precursors, cationic surfactants, organic solvents, and hydrothermal aging, the proposed approach substantially reduces the environmental and economic burdens commonly associated with conventional MSN production while maintaining precise control over textural properties.

Systematic modulation of polysorbate 80 concentration enabled the formation of hollow mesostructured particles with tunable size, high specific surface areas (up to 551.5 m^2^ g^−1^), large pore volumes (up to 1.98 cm^3^ g^−1^), and well-developed mesoporosity. The optimized MSN exhibited the most favorable physicochemical characteristics and demonstrated efficient amoxicillin (AMX) removal from aqueous media. The adsorption process was favored under mildly acidic conditions (pH 5.0) and was accurately described by the pseudo-second-order kinetic and Langmuir isotherm models, indicating adsorption dominated by surface-controlled interactions on energetically homogeneous sites, with a maximum monolayer adsorption capacity of 91.3 mg g^−1^. Moreover, nanosorbent maintained approximately 94% of its initial adsorption performance after five regeneration cycles and achieved AMX recovery efficiencies of 81–89% in spiked tap water and synthetic municipal wastewater, highlighting its robustness in complex water matrices.

These findings demonstrate clearly that sodium silicate-derived MSNs can achieve adsorption performances comparable to those reported for materials synthesized from conventional alkoxysilane precursors while offering notable advantages in sustainability, cost efficiency, and process simplicity. The synergistic combination of high surface area, accessible mesoporosity, abundant surface silanol groups, and structural stability renders these materials promising candidates for environmental remediation and other interfacial applications, including catalysis and drug delivery. Future investigations should focus on process scale-up, techno-economic and life-cycle assessments, and targeted surface functionalization to enhance selectivity toward a broader range of emerging contaminants in real water systems.

## Figures and Tables

**Table 1:** BET specific surface area, BJH pore diameter, and total pore volume of the synthesized MSNs.

Sample	Surface Area (m^2^ g^−1^)	Pore Diameter (nm)	Pore Volume (cm^3^ g^−1^)
MSN-1	264.9	14.83	0.98
MSN-2	309.8	18.78	1.45
MSN-3	551.5	14.33	1.98

**Table 2:** Kinetic model equations and fitting parameters for AMX adsorption onto MSN-3.

Model	Equation	Obtained Parameters	R^2^	RMSE
Pseudo-first-order (PFO)	*q_t_* = *q_e_* (1 − *e*^−*k*^_1_^*t*^)	*q_e_* = 58.2 mg g^−1^; *k*_1_ = 0.018 min^−1^	0.937	3.23
Pseudo-second-order (PSO)	*q_t_* = *k*_2_ *q_e_*^2^ *t*/(1 + *k*_2_ *q_e_* *t*)	*q_e_* = 66.7 mg g^−1^; *k*_2_ = 3.9 × 10^−4^ g mg^−1^ min^−1^	0.995	0.89

**Table 3:** Isotherm model equations and fitting parameters for AMX adsorption onto MSN-3.

Model	Equation	Obtained Parameters	R^2^	RMSE
Langmuir	*q_e_* = *q_m_* *K_l_* *C_e_*/(1 + *K_l_* *C_e_*)	*q_m_* = 91.3 mg g^−1^; *K_l_* = 0.10 L mg^−1^	0.97	5.44
Freundlich	*q_e_* = *K_F* *C_e_*^(1/*n*)	*K_F* = 18.7; *n* = 2.4	0.88	9.75

**Table 4:** Recovery of AMX from spiked real water samples using MSN-3 (*n* = 3).

Scheme	Spiked (mg L^−1^)	Found (mg L^−1^)	Recovery (%)	RSD (%)
Tap water	10	8.85 ± 0.36	88.5	4.06
Tap water	50	42.20 ± 1.65	84.4	3.91
Synthetic municipal wastewater [57]	10	8.35 ± 0.40	83.5	4.79
Synthetic municipal wastewater [57]	50	40.52 ± 1.70	81.04	4.20

%RSD: relative standard deviation. Concentrations are expressed as mean ± SD (*n* = 3).

## Data Availability

The data that support the findings of this study are available from the corresponding author upon reasonable request.
